# A phase I study of ontuxizumab, a humanized monoclonal antibody targeting endosialin, in Japanese patients with solid tumors

**DOI:** 10.1007/s10637-018-0713-7

**Published:** 2019-01-09

**Authors:** Toshihiko Doi, Takeshi Aramaki, Hirofumi Yasui, Kei Muro, Masafumi Ikeda, Takuji Okusaka, Yoshitaka Inaba, Kenya Nakai, Hiroki Ikezawa, Ryo Nakajima

**Affiliations:** 1 National Cancer Center Hospital East, Chiba, Japan; 2 Shizuoka Cancer Center, 1007 Shimonagakubo Nagaizumi-cho, Sunto-gun, Shizuoka, 411-8777 Japan; 3 Aichi Cancer Center Hospital, Aichi, Japan; 4 National Cancer Center Hospital, Tokyo, Japan; 5 Eisai Co. Ltd., Tokyo, Japan

**Keywords:** Ontuxizumab, Endosialin, Monoclonal antibody, CD248 antigen inhibitor, Phase I

## Abstract

*Background* We conducted a first-in-Japanese, phase I study of ontuxizumab, a humanized, anti-endosialin monoclonal antibody, to confirm its tolerability, safety, and pharmacokinetics, and identify exploratory efficacy. *Methods* This was a multicenter, multiple-dose, open-label study in Japanese patients aged ≥20 years with solid tumors, including gastric cancer (GC) or advanced hepatocellular carcinoma (HCC), who had failed standard chemotherapy. The study comprised two parts: part 1 (dose-escalation; ontuxizumab 2–12 mg/kg weekly) and part 2 (cohort-expansion; 4 or 8 mg/kg weekly, or 12 mg/kg biweekly). *Results* Fifteen patients were treated in part 1, and 31 in part 2 (16 patients with GC and 15 with HCC). In part 1, the most common treatment-related, treatment-emergent adverse event (TEAE) was fatigue (20%); no patients had grade ≥ 3 treatment-related TEAEs. In part 2, the most common treatment-related TEAEs were constipation, malaise, hiccups, and increased bilirubin; treatment-related grade 3 TEAEs occurred in two patients with HCC. In part 1, no patients achieved a partial response, and 6/15 (40%) had stable disease (SD). In part 2, 2/15 patients (13.3%) with GC and 8/15 (53.3%) with HCC had SD. Tumor shrinkage was observed in 5/15 HCC patients (33.3%). *Conclusions* Ontuxizumab, up to a dosage of 12 mg/kg weekly, was generally safe and well tolerated in this population, with no dose-limiting toxicities. The maximum tolerated dose was not reached; 8 mg/kg weekly or 12 mg/kg biweekly were the recommended dosages. We observed long-term disease stabilization in GC and extraskeletal chondrosarcoma, and tumor shrinkage in gastrointestinal stromal tumor and HCC. **Trial registration:** NCT01773434 (ClinicalTrials.gov).

**Electronic supplementary material:**

The online version of this article (10.1007/s10637-018-0713-7) contains supplementary material, which is available to authorized users.

## Introduction

Endosialin (also known as tumor endothelial marker-1 [TEM1/CD248]) is an 80.9 kDa sialic acid-rich transmembrane glycoprotein of the C-type, lectin-like receptor family [1]. It is expressed on the surface of several cells, including fibroblasts, mesenchymal stem cells, and endothelial progenitor cells during embryonic development [1–3]. It is also commonly expressed on host-derived stromal cells, such as pericytes associated with tumor blood vessels and cancer-associated fibroblasts (CAFs), which are thought to play a key role in the development of tumor neovascular networks and stromal interactions [4].

Endosialin mRNA and protein expression have been associated with multiple human cancers, including colorectal, gastric, renal, breast, pancreatic, lung, endometrial, ovarian, and neuroectodermal tumors, hepatocellular carcinoma (HCC), and metastatic malignant melanoma [1, 5–10]. Direct endosialin expression on tumor cells has also been noted in some subsets of sarcoma [11].

Endosialin is implicated in tumor-cell vascular adhesion and migration, neoangiogenesis, local invasion, and metastasis [1, 12–16]. Interaction of endosialin with CAFs in gastric cancer and HCC also plays a role in tumor growth and metastasis [17, 18]. Moreover, endosialin overexpression has been associated with aggressive tumor behavior and poor patient prognosis [8, 19, 20]. Indeed, in a collaborative analysis with Almac Diagnostics, endosialin was expressed to a greater extent in an angio-immune/mesenchymal subgroup than in other patients, thus suggesting that endosialin might play an important role in gifting a mesenchymal profile to cancers.

Although *Tem1* knockout mice showed no obvious phenotype and demonstrated normal wound healing, transplanted tumors grew more slowly, were less invasive, and fewer metastases developed than in wild-type mice [5]. Thus, based on the abovementioned findings, and on other preclinical research [21], endosialin was considered a safe and promising target for anticancer treatment.

Ontuxizumab is a humanized, anti-endosialin, IgG1κ monoclonal antibody with a structure comprising two heavy chains and two light chains with disulfide bonds. In vivo, ontuxizumab significantly affected syngeneic tumor growth and tumor metastasis in human CD248 knock-in mice. Compared with untreated tumors, the blood vessels of ontuxizumab-treated tumors were shortened and distorted. Additionally, CD248 levels on the cell surfaces of neovascular pericytes were significantly reduced due to CD248 internalization. This was accompanied by reduced smooth muscle α-actin expression, depolarization of pericytes and endothelium, and ultimately, dysfunctional microvessels [22].

The first-in-human study of ontuxizumab (MORAb-004-001) was conducted in the US, and was an open-label, phase I trial in patients with solid tumors (without intracranial involvement or metastases) who had failed standard chemotherapy [23]. This study evaluated the safety and pharmacokinetics (PK) of ontuxizumab in patients with solid tumors at doses ranging from 0.0625–16 mg/kg. Dose-limiting toxicities (DLTs) occurred in two patients (grade 3 vomiting) at 16 mg/kg weekly, while no DLTs were reported up to 8 mg/kg weekly; 12 mg/kg weekly was defined as the maximum tolerated dosage.

The present study (MORAb-004-J081–103) is a first-in-Japanese study of ontuxizumab. After confirmation of tolerability, the study expanded cohorts to further characterize the tolerability, safety, and PK of ontuxizumab and to identify the exploratory efficacy and PK of ontuxizumab.

## Methods

### Study design

This was a multicenter, multiple-dose, open-label, phase I study of ontuxizumab in Japanese patients with solid tumors (without intracranial metastases) who had failed standard chemotherapy. The study was conducted in two parts: a dose-escalation portion to assess the tolerability and safety of ontuxizumab monotherapy in patients with solid tumors (study part 1); and a cohort-expansion portion (study part 2), which was designed, in part, to assess the PK relationships of ontuxizumab in patients with gastric cancer or HCC.

Ontuxizumab was administered as an intravenous infusion. The stock solution was 5 mg/mL, which was diluted with normal saline, as required, before administration. In study part 1, patients received weekly administrations of ontuxizumab on days 1, 8, 15, and 22 of a 4-week cycle. The dose started at 2 mg/kg and escalated up to 12 mg/kg. Treatment at the next dose started if no DLTs were observed. In study part 2, each patient at 4 or 8 mg/kg received ontuxizumab infusions on days 1, 8, 15, and 22 of a 4-week cycle. Each patient at 12 mg/kg received biweekly ontuxizumab infusions on days 1 and 15 of a 4-week cycle. Patients repeated this cycle at the same dosage until disease progression, unless they met the discontinuation criteria. In study part 2, three dose levels were examined, based on the safety profile obtained in study part 1: 4 or 8 mg/kg (administered on a weekly basis), and 12 mg/kg (administered once every 2 weeks). Study part 2 consisted of cohort A, which comprised patients with a histologic diagnosis of gastric cancer, and cohort B, which comprised patients with a histologic diagnosis of HCC.

### Study participants

No patients had intracranial involvement or metastases. All patients had failed or were resistant to standard chemotherapy and had no appropriate therapies available. All patients gave written informed consent to participate.

The protocol, informed consent form, and related documents were approved by the relevant Institutional Review Boards. The study was conducted in accordance with Good Clinical Practice guidelines, as outlined in the Principles of the World Medical Association Declaration of Helsinki, and the Pharmaceutical Affairs Law of Japan.

#### Inclusion criteria

Included patients were Japanese males or females, aged ≥20 years, with solid tumors, gastric cancer, or a clinically confirmed diagnosis of advanced HCC (Child-Pugh class A). Exclusion criteria are shown in supplementary methods (Online Resource 1). All patients had Eastern Cooperative Oncology Group (ECOG) performance status 0–1 and adequate organ function. Patients with a preserved tumor biopsy sample taken before entry into the study had to provide written informed consent for the sample to be used.

### Study objectives

The primary study objective was to investigate the tolerability and safety of multiple intravenous infusions of ontuxizumab in Japanese patients with solid tumors (gastric cancer or HCC). Secondary study objectives were to determine the maximum tolerated dose (MTD) of ontuxizumab as defined by DLTs; establish the serum PK of ontuxizumab; detect any anti-drug antibody (ADA) response to multiple intravenous infusions of ontuxizumab; and describe changes in objective measurements of tumor size after treatment with ontuxizumab.

### Study assessments

#### Safety

Safety assessments included monitoring and recording all adverse events (AEs) and serious adverse events (SAEs), various laboratory parameter investigations, and physical examinations.

For DLT evaluation, the severity (grade) of AEs was classified according to the latest version of Common Terminology Criteria for Adverse Events (CTCAE) version 4.0–Japan Clinical Oncology Group (JCOG). A DLT was defined as any grade ≥ 3 hematologic or non-hematologic toxicity (CTCAE version 4.0–JCOG definition) related to ontuxizumab administration, with the following exceptions: grade ≥ 3 anaphylactic or anaphylactoid reactions; infusion-related toxicities that could be treated or controlled to grade ≤ 2 by maximal medical management within 48 h (e.g., fever, chills, nausea, vomiting, or diarrhea controllable with antipyretic, anti-emetic or antidiarrheal agents); abnormal laboratory parameters not requiring medical treatment; and pretreatment grade 2 liver function test abnormalities that progressed to grade 3 during the study, if the reason for progression was considered by the investigators to be the underlying disease and not ontuxizumab. The MTD was the highest dose at which no more than one of six patients experienced a DLT in cycle 1.

#### Pharmacokinetics

Blood samples were collected and serum ontuxizumab and ADA concentrations were measured by electrochemiluminescent immunoassay.

#### Efficacy

Tumor assessment based on Response Evaluation Criteria In Solid Tumors (RECIST) version 1.1 was performed for evaluable tumor lesions. The same modality was used to characterize each identified and reported lesion at baseline and through follow-up, by suitable computed tomography (CT), magnetic resonance imaging (MRI), or alternatives.

The following items were assessed for tumor response based on RECIST: best overall response (BOR) — i.e., the best response recorded from the start of treatment until study end; objective response rate (ORR) — defined as the proportion of patients with a BOR of complete response (CR) or partial response (PR), and disease control rate (DCR) — defined as the proportion of patients with a BOR of CR, PR, or stable disease (SD). To achieve a BOR of SD, measurements at ≥7 weeks after the administration had to meet the SD criteria.

For tumor assessment, CT and MRI with contrast agents were recommended, except in patients with a history of allergy to oral or intravenous contrast agents. If patients were allergic to intravenous iodinated contrast agents, CT with oral contrast agents or, for the abdomen and pelvis, MRI with intravenous, gadolinium-based contrast agents were considered. Low-dose CT images from positron emission tomography CT, CT images used for absorption correction, or ultrasonography were not used for tumor assessment.

A tumor with a longest diameter of ≥10 mm was regarded as a target lesion (or when CT scans had a slice width > 5 mm). In the case of lymph node involvement, a tumor with a shortest diameter of ≥15 mm was regarded as a target lesion.

### Statistical methods

All statistical analyses were performed using SAS software version 9.3 (SAS Institute, Cary, NC, USA). The safety analysis set comprised patients who received at least one administration of the study drug and had ≥1 post-dose safety assessment. Safety analyses other than DLTs were based on the safety analysis set. The number (percent) of patients with treatment-emergent AEs (TEAEs, defined as AEs that emerged, re-emerged, or worsened during treatment, or an AE that occurred from the first dose until 45 days after the last dose) and treatment-emergent SAEs was summarized by Medical Dictionary for Regulatory Activities system organ class and preferred term.

The DLT analysis set comprised patients who registered in study part 1, received at least three administrations of the study drug in cycle 1, and had a DLT assessment. Patients with DLTs were included in the DLT analysis set, regardless of the number of administrations. DLT analyses were based on the DLT analysis set. The number (percent) of patients who experienced DLTs was summarized, and DLTs were also summarized by type.

The PK analysis set comprised patients who received at least one administration of study drug and had sufficient PK data to derive at least one PK parameter. The safety analysis set was used for individual ontuxizumab concentration listings. The PK analysis set was used for summaries of ontuxizumab serum concentrations and summaries and analyses of PK parameters.

Summary statistics were tabulated for serum ontuxizumab concentrations according to study part (part 1, part 2 [Cohort A, Cohort B]), dose, and time point. Linear and semi-log plots of serum ontuxizumab concentration-time profiles for individual patients were displayed by study part and dose group. Using non-compartmental methods (WinNonlin software version 6.4 [Pharsight Corporation Inc., Mountain View, CA, USA]), serum ontuxizumab concentrations were analyzed to determine PK parameters, including peak plasma concentration (C_max_), time to C_max_, area under the plasma concentration-time curve (AUC), clearance, and volume of distribution after single and multiple doses.

The efficacy analysis set comprised patients who received ≥1 administration of study drug and had at least one post-dose efficacy measurement. According to tumor response, based on RECIST version 1.1, BOR was summarized by each dose group, and for all dose groups combined. The percent changes in the sum of diameters of tumor target lesions were summarized using a waterfall plot.

## Results

### Participants

**Fig. 1:**
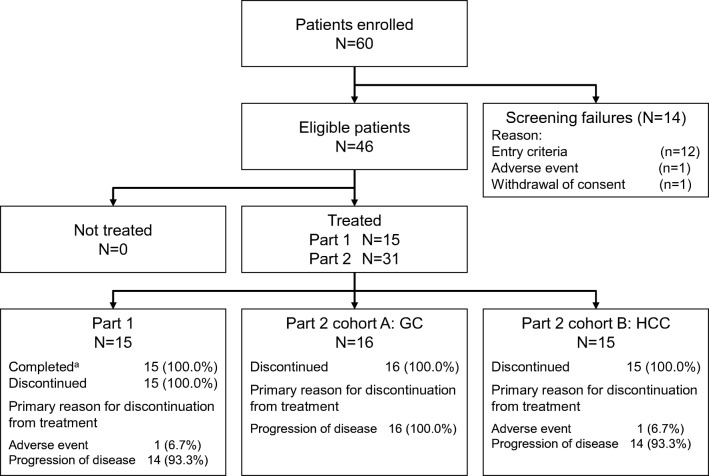
Patient disposition and primary reasons for study discontinuation. ^a^ Patients who received at least three administrations of study drug or developed DLTs during cycle 1. DLT, dose-limiting toxicity; GC, gastric cancer; HCC, hepatocellular carcinoma

A total of 15 patients were treated in study part 1 and 31 were treated in study part 2 (16 with gastric cancer and 15 with HCC) (Fig. 1).

Patient demographic data are shown in Table 1. Mean age was similar for patients in study part 1 versus study part 2. Approximately half of the patients in study part 1 and 80% of patients in study part 2 were male. In study part 2, patients with HCC had a greater mean body weight than those with gastric cancer or solid tumors. Almost all patients had undergone previous surgery and chemotherapy, whereas previous radiotherapy was less common. In study part 2, 58.1% of patients had ECOG performance status 0, and 41.9% had a performance status 1. Further, all patients with HCC had Child-Pugh score of 5–6, and Barcelona Clinic Liver Cancer stage B or C disease.

**Table 1:** Patient demographics

	Part 1: Solid tumor				Part 2: Gastric cancer			Part 2: HCC		
Category	2 mg/kg weekly (*N* = 3)	4 mg/kg weekly (*N* = 3)	8 mg/kg weekly (*N* = 3)	12 mg/kg weekly (*N* = 6)	4 mg/kg weekly (*N* = 5)	8 mg/kg weekly (*N* = 5)	12 mg/kg biweekly (*N* = 6)	4 mg/kg weekly (*N* = 5)	8 mg/kg weekly (*N* = 5)	12 mg/kg biweekly (*N* = 5)
Age (years), mean (SD)	67.7 (4.9)	52.0 (6.1)	64.3 (9.0)	58.8 (12.0)	66.0 (7.7)	67.0 (6.8)	62.2 (14.0)	66.2 (7.9)	70.0 (9.0)	55.6 (12.4)
Sex, male	2	1	1	3	5	5	4	4	3	4
Bodyweight (kg), mean (SD)	55.9 (16.7)	46.9 (1.9)	60.8 (8.2)	65. 5 (10.7)	56.2 (7.3)	54.6 (9.7)	48. 3 (5.9)	69.7 (18.9)	56.0 (14.8)	62.9 (9.7)
ECOG PS										
0	3	2	1	5	1	3	3	3	4	4
1	0	1	2	1	4	2	3	2	1	1
Treatment history										
Previous surgery	3	3	3	5	3	5	4	5	2	5
Previous radiotherapy	1	1	0	2	0	1	0	0	0	3
Previous chemotherapy	3	3	2	6	5	5	6	5	5	5
Primary malignancy										
Gastric cancer	1	1	0	1						
Breast cancer	0	0	0	2						
Gastrointestinal stromal tumor	0	0	0	2						
Adrenocortical cancer	0	1	0	0						
Extraskeletal chondrosarcoma	0	0	1	0						
Hypopharyngeal cancer	0	1	0	0						
Intrahepatic cholangiocarcinoma	0	0	0	1						
Lung cancer	1	0	0	0						
Malignant pheochromocytoma	1	0	0	0						
Renal pelvis cancer	0	0	1	0						
Thymic carcinoma	0	0	1	0						
Child–Pugh score										
5								3	3	4
6								2	2	1
Barcelona Clinic Liver Cancer stage										
Stage B								1	1	0
Stage C								4	4	5

ECOG, Eastern Cooperative Oncology Group; HCC, hepatocellular carcinoma; PS, performance status; SD, standard deviation

All treated patients in study part 1 and study part 2 were included in the safety, DLT, efficacy, and PK analysis sets. All 15 patients in study part 1 completed at least three administrations during cycle 1. One patient (2 mg/kg) discontinued ontuxizumab due to AEs, and 14 discontinued due to disease progression. All 31 patients in study part 2 received at least one dose of ontuxizumab. One patient with HCC (4 mg/kg weekly) discontinued ontuxizumab due to AEs, and the remaining 30 patients discontinued due to disease progression.

### Safety

A total of 46 patients (study part 1, *n* = 15; study part 2, *n* = 31) received ontuxizumab. In study part 1, the median number of cycles was 2.0 (range: 1 to 8), and the median exposure duration was 51.0 days. In study part 2, the median number of cycles was 2.0 (range: 1 to 4) in patients with gastric cancer, and 2.0 (range: 2 to 36) in patients with HCC; median exposure duration was 49.0 days and 51.0 days, respectively. The median percentage of received versus planned doses was 100.0% (range: 75.0%–100.0%).

No treatment-related TEAEs leading to discontinuation from study treatment occurred in study parts 1 and 2. In study part 1, the most frequently reported treatment-related TEAE was fatigue (20.0%; 3/15), followed by constipation, decreased appetite, hyperkalemia, infusion-related reactions, and rash (all with an incidence of 13.3%; 2/15) (Table 2). An infusion-related reaction occurred in one patient in the 4 mg/kg group (dizziness and dry mouth within 3 h after dosing on day 1 that lasted for 18–24 h; study drug interruption was necessary, but the patient recovered without additional intervention), and in one patient in the 12 mg/kg group (pyrexia within 6–9 h after dosing on day 1 that lasted for 3–6 h; no study drug interruption was necessary, and the patient recovered without intervention). In study part 1, no patients had grade ≥ 3 treatment-related TEAEs. No DLTs were observed up to 12 mg/kg in study part 1, and the MTD was not reached.

**Table 2:** Treatment-related adverse events

	Part 1: Solid tumor					Part 2: Gastric cancer				Part 2: HCC				Part 2 total (*N* = 31)
	2 mg/kg weekly (*N* = 3)	4 mg/kg weekly (*N* = 3)	8 mg/kg weekly (*N* = 3)	12 mg/kg weekly (*N* = 6)	Total (*N* = 15)	4 mg/kg weekly (*N* = 5)	8 mg/kg weekly (*N* = 5)	12 mg/kg biweekly (*N* = 6)	Total (*N* = 16)	4 mg/kg weekly (*N* = 5)	8 mg/kg weekly (*N* = 5)	12 mg/kg biweekly (*N* = 5)	Total (*N* = 15)	
Any TEAE	2	2	2	2	8	3	3	2	8	4	3	3	10	18
Fatigue	2	1	0	0	3	0	0	0	0	0	0	0	0	0
Constipation	1	1	0	0	2	1	1	0	2	0	1	0	1	3
Decreased appetite	0	1	1	0	2	0	0	0	0	0	0	0	0	0
Hyperkalemia	1	0	1	0	2	0	0	0	0	0	0	0	0	0
Infusion-related reaction	0	1	0	1	2	0	0	0	0	0	0	0	0	0
Rash	0	0	0	2	2	0	0	0	0	0	0	0	0	0
Malaise	0	0	0	0	0	0	0	1	1	2	0	0	2	3
Hiccups	0	0	0	0	0	0	0	0	0	1	0	2	3	3
Bilirubin increased	0	0	0	0	0	1	0	0	1	1	1	0	2	3
Nausea	0	0	0	0	0	0	0	1	1	0	0	1	1	2
Vomiting	0	0	0	0	0	0	1	1	2	0	0	0	0	2
Pyrexia	0	0	0	0	0	1	0	1	2	0	0	0	0	2
Hypoalbuminemia	0	0	0	0	0	0	0	0	0	1	1	0	2	2
AST increased	0	0	0	0	0	0	0	0	0	1	1	0	2	2
ALT increased	0	0	0	0	0	0	0	0	0	1	1	0	2	2

ALT, alanine aminotransferase; AST, aspartate transferase; HCC, hepatocellular carcinoma; TEAE, treatment-emergent adverse event

In study part 2, the most frequently reported treatment-related TEAEs were constipation, malaise, hiccups, and increased bilirubin (all with an incidence of 9.7%; 3/31), followed by nausea, vomiting, pyrexia, hypoalbuminemia, increased aspartate aminotransferase (AST), and increased alanine aminotransferase (ALT; all with an incidence of 6.5%; 2/31) (Table 2). No investigator-reported AEs of interest were reported in study part 2. Treatment-related grade 3 TEAEs occurred in two patients with HCC (one patient had increased ALT and increased AST in the 4 mg/kg-weekly group, and another had hyperglycemia in the 8 mg/kg-weekly group). No SAEs were reported as treatment-related by the investigator.

### Pharmacokinetics

In study parts 1 and 2, intravenously administered ontuxizumab on cycle 1, day 1, and on cycle 1, day 22 (2 mg/kg, 4 mg/kg, 8 mg/kg, and 12 mg/kg weekly), or cycle 2, day 1 (12 mg/kg biweekly) was eliminated slowly from serum with a long half-life after C_max_ had been attained (Table 3). Mean elimination half-life (t_½_) values increased from 62.6 to 168 h with increasing ontuxizumab dosage from 2 to 12 mg/kg on cycle 1, day 1 (study part 1). In study parts 1 and 2, serum ontuxizumab concentrations reached steady-state approximately 1008 h after the start of repeated-dose administration (at cycle 2).

**Table 3:** Summary of PK parameters for ontuxizumab after infusion at cycle 1, day 1, and cycle 1, day 22

PK parameter	Part 1: Solid tumor				Part 2: Gastric cancer			Part 2: HCC		
	2 mg/kg weekly (*N* = 3)	4 mg/kg weekly (*N* = 3)	8 mg/kg weekly (*N* = 3)	12 mg/kg weekly (*N* = 6)	4 mg/kg weekly (*N* = 5)	8 mg/kg weekly (*N* = 5)	12 mg/kg biweekly (*N* = 6)	4 mg/kg weekly (*N* = 5)	8 mg/kg weekly (*N* = 5)	12 mg/kgbiweekly (*N* = 5)
Cycle 1, day 1										
C_max_ (μg/mL)	41.6 ± 10.9	104 ± 19.3	182 ± 12.5	258 ± 41.1	67.8 ± 10.4	174 ± 39.5	243 ± 32.5	93.9 ± 14.6	181 ± 25.5	282 ± 41.7
t_max_ (h)	1.200 (1.00–1.77)	2.000 (1.07–4.95)	2.380 (1.65–2.55)	2.985 (1.83–4.00)	1.120 (1.08–1.60)	2.000 (1.48–2.03)	1.850 (1.75–5.63)	1.380 (1.03–5.13)	2.100 (1.42–3.65)	2.650 (1.75–6.30)
AUC_(0–24h)_ (μg•h/mL)	802 ± 227	1850 ± 384	3420 ± 656	4740 ± 648	1280 ± 184	3020 ± 800	4380 ± 906	1730 ± 347	3210 ± 484	5200 ± 575
AUC_(0–68h)_ (μg•h/mL)	3000 ± 862	6680 ± 1080	14,300 ± 2520	20,200 ± 2700	4970 ± 675	13,800 ± 4040	–	6290 ± 1120	12,800 ± 2370	–
AUC_(0–336h)_ (μg•h/mL)	–	–	–	–	–	–	26,100 ± 4630	–	–	29,300 ± 1930
AUC_(0–inf)_ (μg•h/mL)	3580 ± 1150	8860 ± 1900^a^	21,200 ± 4030^a^	29,400 ± 3460^a^	6450 ± 1170	22800^d^	31,400 ± 6000^e^	7760 ± 1380^c^	20,400 ± 4300^f^	36,300 ± 4160
t_½_ (h)	62.6 ± 9.78	72.2 ± 14.7^a^	140 ± 55.2	168 ± 70.7	77.9 ± 18.0	154 ± 43.3^c^	156 ± 66.6	89.8 ± 50.0	107 ± 37.0	142 ± 30.7
CL (L/h)	0.0324 ± 0.00931	0.0222 ± 0.00629^a^	0.0220 ± 0.000424^a^	0.0313 ± 0.0100^a^	0.0355 ± 0.00519	0.0197 ^d^	0.0198 ± 0.00468 ^e^	0.0374 ± 0.00991 ^c^	0.0266 ± 0.00794 ^f^	0.0210 ± 0.00262
Vss (L)	2.86 ± 0.407	2.20 ± 0.318^a^	3.48 ± 0.467^a^	4.45 ± 1.18^a^	3.90 ± 0.582	3.27^d^	3.55 ± 0.990^e^	3.71 ± 1.31^c^	3.88 ± 1.14^f^	4.12 ± 0.826
MRT (h)	90.8 ± 13.6	101 ± 14.3^a^	158 ± 18.4^a^	144 ± 7.78^a^	112 ± 25.2	166^d^	182 ± 35.2^e^	98.2 ± 19.7^c^	147 ± 21.0	196 ± 30.8
Cycle 1, day 22 /Cycle 2, day 1										
C_max_ (μg/mL)	51.3 ± 14.8	152 ± 16.5	353 ± 36.6	417 ± 100	92.7 ± 19.9	264 ± 67.5	284 ± 48.1^c^	125 ± 22.6	247 ± 49.2	374 ± 54.2
t_max_ (h)	2.580 (1.50–4.58)	1.520 (1.10–1.63)	1.180 (1.00–5.12)	2.975 (2.40–5.52)	2.550 (1.05–2.73)	1.300 (0.87–2.08)	2.160 (1.95–3.25)^c^	1.620 (1.15–4.72)	1.550 (0.98–2.17)	2.700 (1.77–3.60)
AUC_(0–168h)_ (μg•h/mL)	4020 ± 1350	13,500 ± 1800	29,600 ± 5950	42,700 ± 9150^b^	8710 ± 2210	24,900 ± 7090	–	10,200 ± 1960	20,100 ± 4050	–
AUC_(0–336h)_ (μg•h/mL)	–	–	–	–	–	–	39,700 ± 4840^c^	–	–	42,700 ± 7270
t_½_ (h)	85.3 ± 28.3	219 ± 138^a^	121 ± 5.66^a^	180 ± 85.2^b^	142 ± 25.6^c^	193 ± 144	179 ± 26.2^c^	98.9 ± 25.9^c^	119 ± 23.2	210 ± 113 ^c^

^a^*N* = 2, ^b^*N* = 5, ^c^*N* = 4, ^d^*N* = 1, ^e^*N* = 5, ^f^*N* = 3

AUC, area under the plasma concentration–time curve; CL, clearance; C_max_, peak plasma concentration; MRT, mean residence time; PK, pharmacokinetic; t_½_, elimination half-life; t_max_ time to peak plasma concentration; Vss, volume of distribution at steady-state

**Fig. 2:**
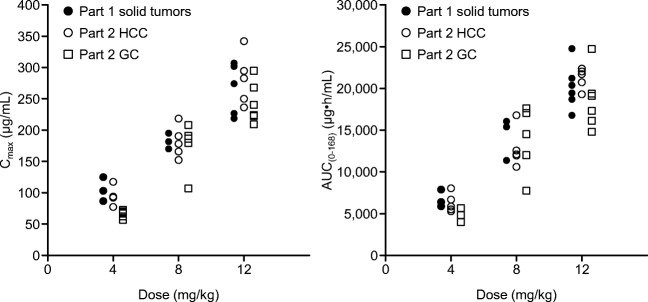
Relationships between ontuxizumab dosage and peak plasma concentration (C_max_) and between dosage and area under the plasma concentration versus time curve (AUC; cycle 1, day 1)**.** Although C_max_ at 4 mg/kg in patients with gastric cancer (part 2) was relatively lower than in patients with solid tumors (part 1) and in patients with HCC (part 2), there were no clinically significant differences between the three groups at all ontuxizumab dosages. GC, gastric cancer; HCC, hepatocellular carcinoma

In study part 1, C_max_ and AUC_(0–168h)_ at both cycle 1, day 1 and cycle 1, day 22 increased in an almost dose-proportional manner (Fig. 2). In study part 2, mean values of C_max_ after weekly administration of ontuxizumab 4 mg/kg and 8 mg/kg on cycle 1, day 22, and after biweekly administration of 12 mg/kg on cycle 2, day 1, were 92.7, 264.0, and 284.0 μg/mL in patients with gastric cancer; and 125.0, 247.0, and 374.0 μg/mL in patients with HCC, respectively. Mean values of AUC_(0–168h)_ after weekly administration of ontuxizumab 4 mg/kg and 8 mg/kg at cycle 1, day 22 were 8710 and 24,900 μg•h/mL in patients with gastric cancer, and 10,200 and 20,100 μg•h/mL in patients with HCC. Mean values of AUC_(0–336h)_ after biweekly administration of ontuxizumab 12 mg/kg on cycle 2, day 1 were 39,700 and 42,700 μg•h/mL in patients with gastric cancer and HCC, respectively.

In study part 2, the mean value of C_min_ on cycle 2, day 15 for ontuxizumab 12 mg/kg biweekly administration was between the trough concentrations of 4 mg/kg and 8 mg/kg weekly administration in patients with gastric cancer or HCC.

### Efficacy

In study part 1, no patients achieved a BOR of a PR. Six of 15 patients (40%) had a BOR of SD, while the remaining nine had progressive disease. Tumor stabilization or shrinkage was observed in patients with gastric cancers and extraskeletal chondrosarcoma, while tumor shrinkage was observed in one patient with gastrointestinal stromal tumor (GIST) (Online Resource 2). The DCR was 40% (Table 4).

**Table 4:** Tumor response (efficacy analysis set)

Category	Part 1: Solid tumor					Part 2: Gastric cancer				Part 2: HCC			
	2 mg/kg weekly (*N* = 3)	4 mg/kg weekly (*N* = 3)	8 mg/kg weekly (*N* = 3)	12 mg/kg weekly (*N* = 6)	Total (*N* = 15)	4 mg/kg weekly (*N* = 5)	8 mg/kg weekly (*N* = 5)	12 mg/kg biweekly (*N* = 6)	Total (*N* = 16)	4 mg/kg weekly (*N* = 5)	8 mg/kg weekly (*N* = 5)	12 mg/kg biweekly (*N* = 5)	Total (*N* = 15)
Best overall response													
Evaluable, *n*	3	3	3	6	15	5	5	5	15	5	5	5	15
Complete response (CR), n	0	0	0	0	0	0	0	0	0	0	0	0	0
Partial response (PR), n	0	0	0	0	0	0	0	0	0	0	0	0	0
Stable disease (SD), n (%)	2 (66.7)	1 (33.3)	2 (66.7)	1 (16.7)	6 (40.0)	0	1 (20.0)	1 (20.0)	2 (13.3)	4 (80.0)	2 (40.0)	2 (40.0)	8 (53.3)
Progressive disease (PD), n (%)	1 (33.3)	2 (66.7)	1 (33.3)	5 (83.3)	9 (60.0)	5 (100.0)	4 (80.0)	4 (80.0)	13 (86.7)	1 (20.0)	3 (60.0)	3 (60.0)	7 (46.7)
Not evaluable (NE), n	0	0	0	0	0	0	0	0	0	0	0	0	0
Not determined, n^a^	0	0	0	0	0	0	0	1	1	0	0	0	0
Objective response rate (CR + PR), n^b^	0	0	0	0	0	0	0	0	0	0	0	0	0
Disease control rate (CR + PR + SD ≥ 7 weeks), n (%)^b^	2 (66.7)	1 (33.3)	2 (66.7)	1 (16.7)	6 (40.0)	0	1 (20.0)	1 (20.0)	2 (13.3)	4 (80.0)	2 (40.0)	2 (40.0)	8 (53.3)

^a^A patient who had no post-baseline tumor assessments due to early discontinuation

^b^Percent values are based on the patients categorized as ‘evaluable’ for each parameter

**Fig. 3:**
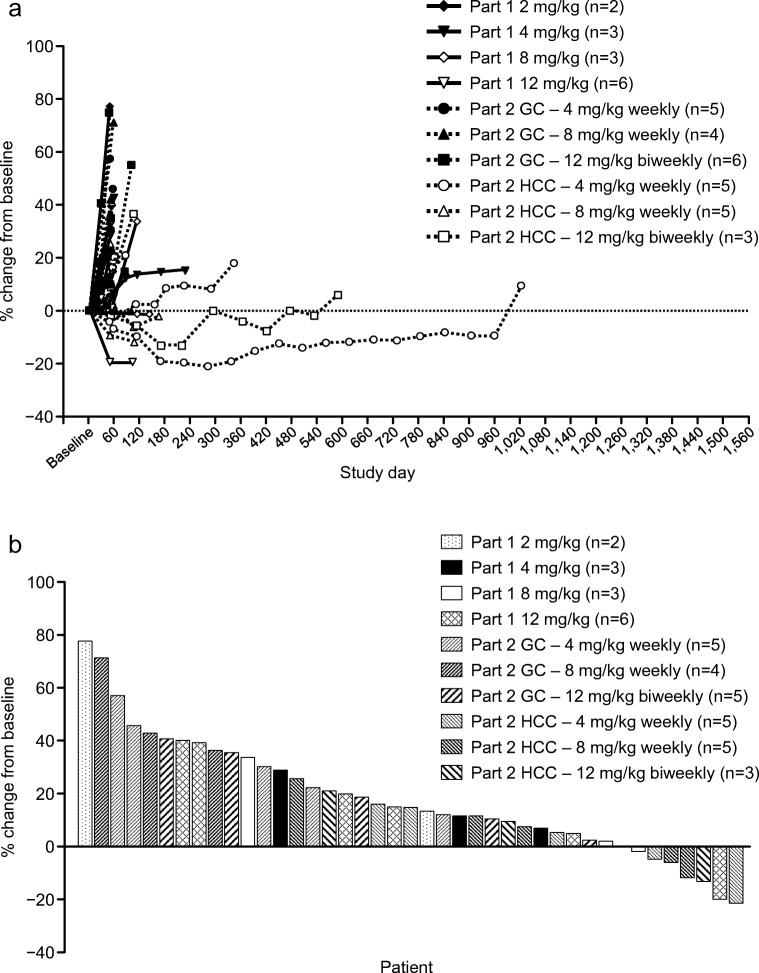
Percent change from baseline in the sum of tumor diameters (efficacy analysis set): **(a)** change over time by patient; and (**b**) waterfall plot of change from baseline to maximum tumor shrinkage. GC, gastric cancer; HCC, hepatocellular carcinoma

In study part 2, one patient with gastric cancer receiving ontuxizumab 12 mg/kg had early discontinuation; therefore, 15 patients with gastric cancer and 15 with HCC were evaluated. Two of 15 patients (13.3%) with gastric cancer and 8 of 15 (53.3%) with HCC had a BOR of SD. The DCR was 13.3% in patients with gastric cancer, and 53.3% in those with HCC (Table 4). Tumor shrinkage was observed in five of 15 HCC patients (33.3%). Preliminary signs of antitumor activity were observed, particularly in patients with HCC, from the percent change in the sum of tumor diameters (Fig. 3a, b).

In patients with HCC in study part 2, non-vascular stromal cells (11/15), perivascular cells (8/15), capillary endothelial cells (7/15), and lymphatic endothelial cells (5/15) showed endosialin expression. Conversely, gastric cancers in study parts 1 and 2 expressed endosialin in perivascular cells (12/17), capillary endothelial cells (10/17), non-vascular stromal cells (9/17), lymphatic endothelial cells (8/17), and endothelial cells (1/17).

## Discussion

This multiple dose, open-label, phase I study was the first to examine the safety and tolerability of ontuxizumab in Japanese patients with solid tumors who had failed standard chemotherapy; an associated aim was to determine the recommended ontuxizumab dosage for future clinical trials. The study was conducted in two parts: a dose-escalation phase (study part 1), and an expansion phase (study part 2).

### Tolerability and safety

Ontuxizumab was generally well tolerated in Japanese patients with solid tumors, up to a dosage of 12 mg/kg weekly, and in Japanese patients with gastric cancer and HCC, across the dosages studied in study part 1. No DLTs were observed up to 12 mg/kg weekly; therefore, the MTD was not reached. A dosage of 12 mg/kg weekly, the MTD of the previous phase I study [23], was also tolerable for Japanese patients.

In study part 1, the most common drug-related TEAE was fatigue, while in study part 2, the most frequent TEAEs were vomiting, constipation, and pyrexia in patients with gastric cancer, and hiccups in patients with HCC. These results are consistent with previous findings from phase I and II studies in non-Japanese patients [23, 24]. No apparent trends were observed in the incidences of TEAEs or treatment-related TEAEs as ontuxizumab dosage increased.

Although one patient had a positive ADA response during the study, this was transient and not associated with significant safety concerns. Moreover, no treatment-related SAEs were reported.

### Pharmacokinetics

Systemic exposure to ontuxizumab, as assessed by C_max_ and AUC_(0–168h)_, increased in an approximately dose-proportional manner on cycle 1, day 1 and cycle 1, day 22 across the dose range of 2–12 mg/kg. After multiple-dose administration, t_½_ values increased with increasing dosage, thus indicating slow clearance of ontuxizumab, especially at higher dosages.

Although data are limited, saturable clearance may play a role in ontuxizumab PK. Indeed, after administration of ontuxizumab 2, 4, 8, and 12 mg/kg weekly on cycle 1, day 22, C_max_ and AUC_(0–168h)_ values in the present study were similar to values in the US study of ontuxizumab in non-Japanese patients [23]. Thus, no differences in ontuxizumab PK profile appear to exist between Japanese and non-Japanese patients.

In study part 2 of the present study, no clinically significant differences in C_max_, AUC_(0–168h)_, or AUC_(0–336h)_ were observed between patients with gastric cancer and those with HCC. This is in contrast to previous studies of trastuzumab and bevacizumab, in which median values for AUC, C_max_, and C_min_ at steady state were approximately 30–40% lower in patients with gastric rather than other cancers [25, 26]. The reasons for these differences are unknown, but such differences suggest an advantage for ontuxizumab, particularly in the treatment of gastric cancer.

Regarding the difference in ontuxizumab dosing regimen between weekly and biweekly administration in study part 2, the value of C_min_ on cycle 2, day 15 for 12 mg/kg biweekly dosing was within the range of the 4 mg/kg and 8 mg/kg weekly dosing schedules in patients with gastric cancer or HCC. The ontuxizumab serum trough concentration with 12 mg/kg biweekly administration was between the trough concentrations for the 4 mg/kg and 8 mg/kg weekly schedules (data not shown). Additionally, the clearance values at dosages of 8 mg/kg weekly and 12 mg/kg biweekly in patients with gastric cancer were 0.0197 and 0.0198 L/h, respectively. Similarly, the clearance values were 0.0266 and 0.0210 L/h, respectively, in patients with HCC. These results demonstrate that clearance was saturated at dosages above 8 mg/kg weekly (Table 3). Therefore, 8 mg/kg weekly was identified as the minimum dosage with saturable clearance, and 12 mg/kg biweekly dosing with ontuxizumab may be clinically meaningful.

### Efficacy

In study part 1, six of 15 patients (40.0%) had a BOR of SD. Tumor stabilization was observed, especially in patients with gastric cancer, extraskeletal chondrosarcoma, or GIST, from the percent change in the sum of longest tumor diameters. In part 2, although complete or partial responses were not observed, tumor shrinkage occurred in five of 15 patients with HCC.

A previous examination of 50 human tumor cell lines and 250 clinical specimens of human cancer, including 20 cancer subtypes, revealed that endosialin was expressed in tumor cells, perivascular cells, and stromal cells in sarcoma; further investigation with 11 types of carcinoma showed that endosialin expression originated from perivascular and stromal cells, and not from carcinoma cells [11]. In our study, both gastric cancer and HCC showed endosialin expression mainly on perivascular and stromal cells and no endosialin expression on carcinoma cells, which is consistent with previous findings. Indeed, a study with frozen tissue specimens found that all gastric cancer specimens (7/7) had endosialin-positive vasculature and stromal cells, whereas no tumor specimens (0/7) had endosialin-positive tumor cells [11].

In a previous examination of gastric cancer tissue samples, CAF-endosialin positivity and CAF-endosialin intensity were significantly correlated with several clinicopathologic factors; moreover, both a higher positive rate and a stronger intensity of endosialin expression in CAFs were associated with poorer recurrence-free survival, cancer-related survival, and overall survival [27]. Recently approved antibodies for the monotherapy management of gastric cancer include ramucirumab (second-line or later) and nivolumab (third-line or later), which showed median progression-free survival (PFS) times of 2.10 months and 1.61 months, respectively [28, 29]. In the current study, tumor stabilization occurred in some gastric cancer patients with an ontuxizumab treatment duration of ≥2 months, suggesting that endosialin inhibition could be a new therapeutic strategy for gastric cancer by targeting endosialin expression in CAFs.

Endosialin expressed on hepatic stromal cells, such as hepatic stellate cells (HSCs), was previously implicated in the initiation and progression of liver metastatic cancers and/or HCC tumors. However, a recent examination with HSCs and an HCC tumor cell co-culture system reported that endosialin-expressing and fully activated HSCs impaired HCC tumor growth, suggesting an inverse causal relationship between HSC-expressed endosialin and HCC growth [30]. Conversely, our study showed tumor shrinkage in some HCC patients treated with ontuxizumab. In addition, the DCR ratio for ≥3 months was 53.3%, which is almost equivalent to that for regorafenib, which was recently approved as monotherapy for the second-line treatment of HCC after demonstrating a median PFS of 3.1 months and DCR of 65% [31]. The mechanism of endosialin action in HCC remains controversial and further studies are needed to elucidate the exact process of ontuxizumab function in HCC.

## Conclusions

Ontuxizumab, up to a dosage of 12 mg/kg weekly, was generally safe and well tolerated in Japanese patients with solid tumors. There were no DLTs, and the MTD was not reached. No clinically significant differences in PK parameters were evident between patients with gastric cancer and those with HCC. According to the safety and PK results of the present study, 8 mg/kg weekly or 12 mg/kg biweekly are the recommended dosages for future studies. Long-term disease stabilization was observed in gastric cancer and extraskeletal chondrosarcoma, and tumor shrinkage was noted in GIST and HCC. All these conditions were resistant and progressive after standard chemotherapies.

## Electronic supplementary material

ESM 1 (DOCX 18 kb)

ESM 2 (DOCX 67 kb)
