# Supplementary material for: A phase I study of ontuxizumab, a humanized monoclonal antibody targeting endosialin, in Japanese patients with solid tumors
Source: Invest New Drugs. 2019 Jan 9;37(5):1061–74. doi: 10.1007/s10637-018-0713-7 (PMC6736902; doi:10.1007/s10637-018-0713-7)
Supplement: Supplementary file 1 — (DOCX 18 kb) [file 10637_2018_713_MOESM1_ESM.docx]

**Title:** A phase I study of ontuxizumab, a humanized monoclonal antibody targeting endosialin, in Japanese patients with solid tumors

**Journal:** Investigational New Drugs

**Authors:** Toshihiko Doi^1^ • Takeshi Aramaki^2^ • Hirofumi Yasui^2^ • Kei Muro^3^ • Masafumi Ikeda^1^ • Takuji Okusaka^4^ • Yoshitaka Inaba^3^ • Kenya Nakai^5^ • Hiroki Ikezawa^5^ • Ryo Nakajima^5^

**Corresponding author:** Takeshi Aramaki; 1007 Shimonagakubo Nagaizumi-cho, Sunto-gun, Shizuoka 411-8777, Japan

Tel: +81-55-989-5222; Fax: +81-55-989-5634; E-mail: t.aramaki@scchr.jp

**Online resource – Online only**

**Online Resource 1** Supplementary methods

*Exclusion criteria*

- Clinically significant cardiovascular disease (e.g., NYHA class III–IV congestive heart failure, uncontrolled angina, or myocardial infarction within 6 months of study entry).
- 12-lead ECG demonstrating clinically significant arrhythmias, including marked baseline prolongation of QT/QTc interval (e.g., repeated demonstration of a QTc interval >500 ms); patients with chronic atrial arrhythmia were eligible to participate.
- Scheduled for surgery with a large open wound.
- A clinically significant hemorrhagic event or history (e.g., intracerebral hemorrhage within 6 months of study entry).
- Chronic systemic anticoagulant therapy.
- Positive serology for HIV.
- Active serious systemic disease, including active bacterial or fungal infection.
- Severe chronic inflammatory disorders (e.g., inflammatory bowel disease).
- Severe lung disease.
- Evidence of an immune or allergic reaction or documented human anti-human antibodies during previous monoclonal antibody treatment.
- Unrelated conditions requiring active antibiotic therapy, cytotoxic therapy (e.g., methotrexate for rheumatoid arthritis), or chronic daily systemic corticosteroid therapy for >4 weeks.
- Large ascites or pleural effusion requiring drainage (≥500 mL) based on results of the most recent CT scan.
- Central nervous system tumor involvement.
- Other active invasive malignancies.
- Previous psychiatric disorders (e.g. alcohol or drug dependence) and patients considered ineligible by the investigator.
- Pregnancy or lactation.
