# Supplementary material for: A phase I study of ontuxizumab, a humanized monoclonal antibody targeting endosialin, in Japanese patients with solid tumors
Source: Invest New Drugs. 2019 Jan 9;37(5):1061–74. doi: 10.1007/s10637-018-0713-7 (PMC6736902; doi:10.1007/s10637-018-0713-7)
Supplement: Supplementary file 2 — (DOCX 67 kb) [file 10637_2018_713_MOESM2_ESM.docx]

**Title:** A phase I study of ontuxizumab, a humanized monoclonal antibody targeting endosialin, in Japanese patients with solid tumors

**Journal:** Investigational New Drugs

**Authors:** Toshihiko Doi^1^ • Takeshi Aramaki^2^ • Hirofumi Yasui^2^ • Kei Muro^3^ • Masafumi Ikeda^1^ • Takuji Okusaka^4^ • Yoshitaka Inaba^3^ • Kenya Nakai^5^ • Hiroki Ikezawa^5^ • Ryo Nakajima^5^

**Corresponding author:** Takeshi Aramaki; 1007 Shimonagakubo Nagaizumi-cho, Sunto-gun, Shizuoka 411-8777, Japan

Tel: +81-55-989-5222; Fax: +81-55-989-5634; E-mail: t.aramaki@scchr.jp

**Online Resources – Online only**

**Online Resource 2** Characteristics of patients achieving tumor stabilization or regression with ontuxizumab

| Patient no. | Primary diagnosis | Age (years) | Previous systemic therapies | Dose of ontuxizumab (mg/kg) | Response to ontuxizumab | Percent change of tumor shrinkage from baseline | Duration of treatment (days)^a^ |
| --- | --- | --- | --- | --- | --- | --- | --- |
| 10011201 | Gastric | 55 | 1. TS-1^a^ (adjuvant) 2. Cisplatin + irinotecan 3. Paclitaxel 4. Docetaxel | 4 mg/kg weekly | SD | NA | 218 |
| 10012211 | Gastric | 61 | 1. Cisplatin + TS-1^a^ 2. Cisplatin + irinotecan 3. Paclitaxel | 8 mg/kg weekly | SD | NA | 91 |
| 10042313 | Gastric | 37 | 1. Cisplatin + TS-1^a^ + trastuzumab 2. Paclitaxel + ramucirumab 3. Investigational drug 4. Irinotecan + trastuzumab 5. FOLFOX^a^ | 12 mg/kg biweekly | SD | NA | 70 |
| 10011302 | Extraskeletal chondrosarcoma | 73 | No treatment | 8 mg/kg weekly | SD | NA | 133 |
| 10011402 | GIST^a^ | 65 | 1. Imatinib 2. Sunitinib 3. Investigational drug | 12 mg/kg weekly | SD | −20.0% | 90 |
| 10012122 | HCC^a^ | 74 | 1. Sorafenib 2. Investigational drug 3. Investigational drug | 4 mg/kg weekly | SD | −4.9% | 328 |
| 10012123 | HCC^a^ | 64 | 1. Sorafenib 2. Investigational drug 3. Investigational drug | 4 mg/kg weekly | SD | −21.6% | 1016 |
| 10022221 | HCC^a^ | 63 | 1. Sorafenib 2. Investigational drug | 8 mg/kg weekly | SD | −12.0% | 99 |
| 10032222 | HCC^a^ | 64 | 1. Sorafenib | 8 mg/kg weekly | SD | −5.8% | 161 |
| 10032321 | HCC^a^ | 59 | 1. Sorafenib 2. Investigational drug | 12 mg/kg biweekly | SD | −13.5% | 583 |

^a^ FOLFOX, leucovorin + 5-fluorouracil + oxaliplatin; GIST, gastrointestinal stromal tumor; HCC, hepatocellular carcinoma; TS-1, tegafur + gimeracil + oteracil potassium
